# Q&A: Trash talk: disposal and remote degradation of neuronal garbage

**DOI:** 10.1186/s12915-018-0487-6

**Published:** 2018-01-30

**Authors:** Meghan Lee Arnold, Ilija Melentijevic, Anna Joelle Smart, Monica Driscoll

**Affiliations:** Department of Molecular Biology and Biochemistry, Rutgers, The State University of New Jersey, A232 Nelson Biological Laboratories, 604 Allison Road, Piscataway, NJ 08855 USA

## Abstract

*Caenorhabditis elegans* neurons have recently been found to throw out cellular debris for remote degradation and/or storage, adding an “extracellular garbage elimination” option to known intracellular protein and organelle degradation pathways. This Q&A describes initial insights into the biology of seemingly selective protein and organelle elimination by challenged neurons, highlighting mysteries of how garbage is distinguished and sorted in the sending neuron, how the garbage-filled “exophers” appear to elicit degradative responses as they transit neighboring tissue, and how non-digestible materials get thrown out of cells again via processes that may be highly relevant to human neurodegenerative disease mechanisms.

## Don’t we already have a good understanding of protein/organelle degradation strategies?

Surprisingly, no. A major theme in the maintenance of cell health is that proteins, which make up about 20% of the cell by weight, must be folded properly for both their own functionality and for preventing misfolded or aggregated proteins from gumming up the works in ways that interfere with other cell activities. Considerable characterization of cell strategies for accomplishing protein quality control has defined chaperone functions (protein folding helpers), the ubiquitin proteasome system (which degrades proteins that are tagged as misfolded or otherwise impaired and ready for destruction), and the autophagy system (which degrades cellular entities including proteins and organelles by targeting defective species to the lysosome for import and degradation) as major cell-intrinsic pathways that keep a cell’s overall protein content in good shape.

Still, this multi-pronged system can become overwhelmed. For example, aging is associated with a decline in efficacy of protein quality control and an increase in aggregation across phyla. Moreover, a striking common feature of late onset neurodegenerative disease is the accumulation of protein aggregates in affected brain tissue. Recent research on neurodegeneration suggests that mysterious mechanisms of protein aggregate management might be added to the internal degradation systems we know about: aggregating species from one neuron can be found to transfer out of that neuron into neighboring cells. Might there be another option for neurons to control their internal protein quality—by throwing out their aggregated proteins? Could cells rely on their neighbors for remote garbage management? Could such a process become dysregulated with age or disease?

## Can neurons really package and throw out contents like trash?

**Fig. 1.**
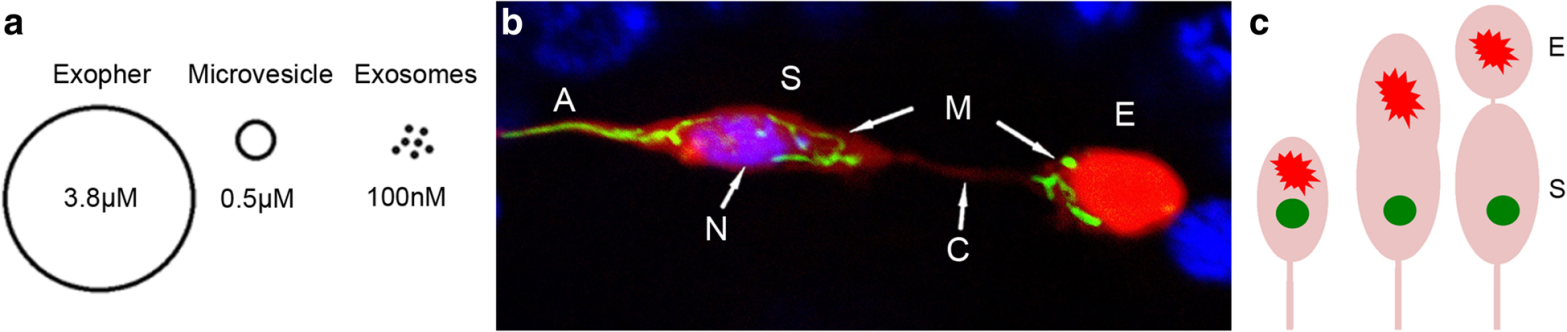
Neurons can sort and throw out the trash. **a** Exophers are large compared to exosomes and microvesicles. **b** Organelles can be extruded in exophers. Mitochondrially-localized green mito-GFP (*M*) and non-targeted red mCherry can be seen in the ALMR neuron (*S* soma, *E* exopher). The soma stains with DAPI nuclear DNA strain (*N*, *purple*), but the exopher does not, supporting exophers are not the outcome of classic cell division. Some mitochondria and most of the mCherry are localized to the exopher compartment, which is attached by a thin connecting tube (*C*); *A* axon; nearby nuclei are *blue*. **c** Cartoon summarizing how a neuron that expresses both aggregating mCherry (*red*) and soluble GFP (*green*) fluorescent markers can preferentially sort most of the red aggregating protein to the exopher (*E*), while retaining most of the soluble GFP in the soma (*S*). Note that as is clear in the real image in **b**, some mCherry can be retained in the soma, though levels usually appear less than in the extruded exopher

It seems like it—-this process has recently been observed in vivo in real time by following fluorescently tagged aggregating proteins in *C. elegans*. We showed that some *C. elegans* neurons can extrude very large membrane-bound vesicles that can selectively include expressed aggregating protein [1]. The easily visualized mechanosensory touch receptor neurons sequester aggregates and send out large ~ 4 μm packages (Fig. 1a) that in some instances can eliminate substantial aggregate concentrations from the soma (Fig. 1b, c). The large extruded membrane-surrounded aggregate-vesicle has been named “exopher” (exo = out; pher = bring). The dramatic process of producing this “trash bag” involves: 1) the polarized localization of most aggregating material to one region in the soma; 2) the formation of a large bud that includes a high concentration of aggregate (the bud can be nearly as large as the soma); 3) the movement of the exopher away from the neuronal soma, while often maintaining a thin tube connection that can still pass material such as fluorescently tagged proteins; and finally 4) breaking of the thin tube connection to dissociate the exopher as it moves further away from the sending neuronal soma. The process can take one to several hours. Note that spread of prions [2] and alpha-synuclein fragments [3] has also been documented in *C. elegans*; it is the visualization of the dynamic extrusion process that is new.

## Exopher? Did you mean to say exosome?

No. Exosomes are tiny extracellular vesicles, typically ~ 40 to 100 nm in diameter (Fig. 1a), that are released in an ESCRT-dependent mechanism following fusion of multivesicular endosomes and storage vacuoles with the plasma membrane [4]. In contrast, *C. elegans* exophers average ~ 4 μm in diameter, and thus are about 100 times larger than exosomes. The biogenesis of exophers involves outward budding of a membrane-bound section of the soma, and does not appear to require the ESCRT protein genes that act in exosome formation. The exopher surface also lacks phosphatidylserine, which further distinguishes it from exosomes and apoptotic bodies.

## What sort of material is thrown out in exophers?

One way of modeling neurodegenerative disease conditions in *C. elegans* is to express human disease proteins in specific neurons. For example, human Huntington’s disease is caused by an expansion in the number of glutamine residues in Huntingtin protein. Expressing a protein that encodes the first 57 amino acids of human Huntingtin linked to an expanded polyQ tract of 128 residues creates neurotoxic aggregates; adding a fluorescent tag such as CFP enables aggregate fate to be followed in the transparent living *C. elegans*. CFP-tagged polyQ aggregates can be concentrated, and extruded, in neuronal exophers [1]. The same is true for an aggregating mCherry fluorophore (Fig. 1b, c). Moreover, 1) a neuron identified to have a high aggregate content has a higher chance of later producing an exopher as compared to a neuron with a low aggregate content, and 2) expression of aggregation-prone proteins is correlated with higher exopher production than expression of more soluble proteins (such as the non-toxic shorter expansion polyQ19 or GFP). These data suggest that aggregated proteins are common cargo that can be released from the neuron in exophers.

## What are the physiological factors that elicit exopher production?

**Fig. 2.**
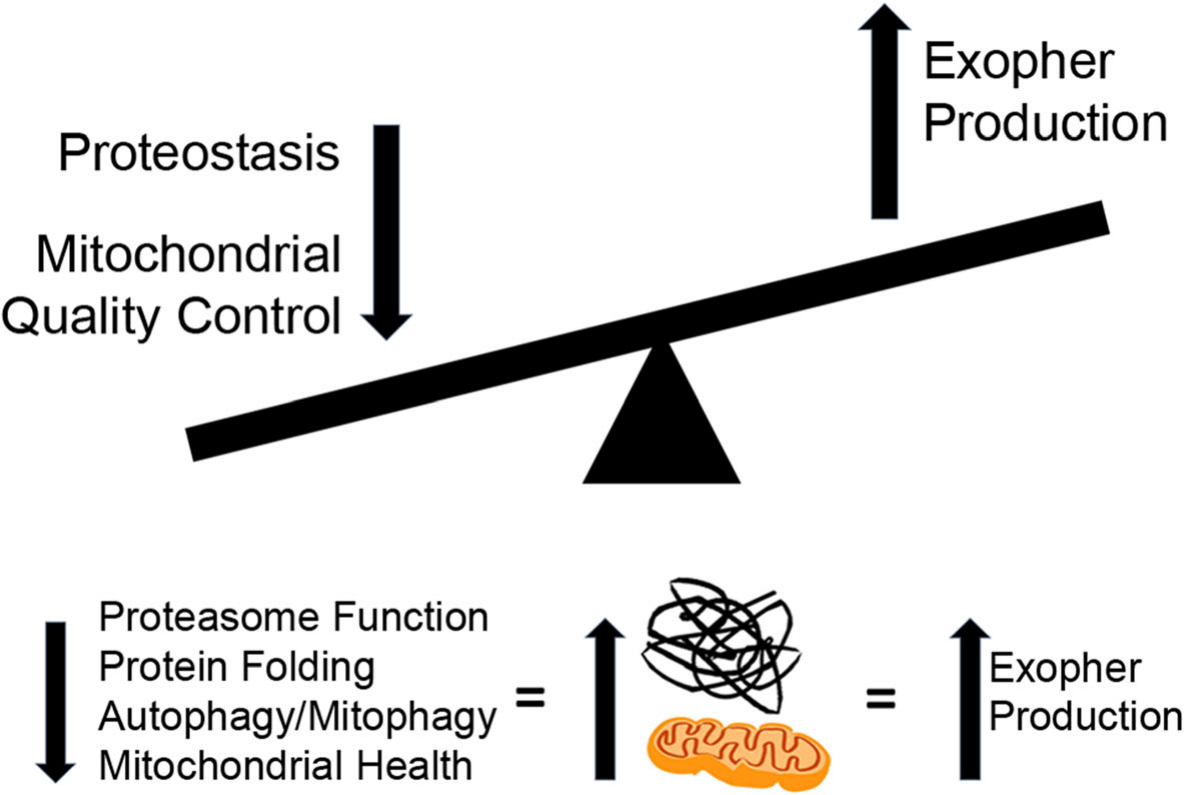
Higher proteostress and impaired mitochondrial quality increase exopher formation. When proteostasis and mitochondrial quality are compromised through protein misfolding, inhibited proteasome function, and/or impaired autophagy and mitophagy, increased protein aggregates and damaged mitochondria are generated and numbers of observed exopher extrusions rise. The suggestion is that increased trash load results in increased external garbage disposal

Experimental manipulations that impair each major branch of known proteostasis pathways increase exopher production (summary in Fig. 2). Genetic disruption of the conserved HSF-1 transcription factor needed for expression of many heat shock factor chaperone folding proteins, RNAi-mediated knockdown of transcripts for multiple genes encoding components of the conserved autophagy pathway, and pharmacological inhibition of the proteasome all increase the number of exophers that are produced by ALMR touch receptor neurons. These observations imply that multiple approaches toward compromising protein homeostasis and elevating the internal junk load can increase the trash extrusion response. What is not yet clear is whether a specific threshold of garbage accumulation needs to be reached to produce an exopher; nor is it clear whether aggregation is more important to exophergenesis than absolute expression level of a foreign protein. Also, although expressing aggregating disease proteins in specific neurons likely induces a cell-autonomous exopher increase, it is not yet clear whether compromising proteo-stress non-autonomously in distant tissues might modulate exopher production in specific neurons.

An important point to be made is that artificially introduced stresses (aggregates or extreme proteostasis perturbations) are not the only conditions that elicit exopher production. For example, when living *C. elegans* are bathed in the non-toxic fluorescent dye Dil, head sensory neurons, which are naturally open to the environment, fill with dye and permit visualization of exopher generation in the absence of egregious transgencially introduced proteo-stress. This suggests that even under “normal” physiological circumstances, presumed to involve native stress levels, neurons can produce exophers. The “natural” exophers are hypothesized to include physiological cellular debris—although contents are currently difficult to study without specific tagging labels or purification of the exopher (purification is not a simple matter—exophers appear to enter the domain of a neighboring cell immediately, so vesicles are not freely released; see below).

## Is only unwanted material thrown out in exophers?

It would seem sensible to only throw trash out, leaving the functional non-aggregated materials behind in the soma to continue executing required neuronal functions. Indeed, if a touch neuron expresses both an aggregating mCherry protein and a simple soluble GFP protein, the exopher it generates contains primarily aggregating mCherry, while the sending soma retains most of the soluble GFP (Fig. 1c). Although one might be concerned that the GFP fluorescence could be preferentially degraded in the exopher compartment, polyQ128CFP and several other GFP tagged proteins can be observed in extruded exophers, suggesting preferential signal degradation is not the reason for the apparent dramatic sorting. This sorting reveals a capacity for the neuron to distinguish what it will throw away from what it will keep. The mechanism for sorting is not known, but can readily be interrogated using *C. elegans* genetic approaches. A reasonable hypothesis is that the aggregates might be recognized by heat shock chaperones and/or ubiquitin ligases and the associated signals may be recognized by motor proteins to initiate compartmentalization and the trip to the “extrusion site” of the cell.

## What else can go into the gigantic exophers?

DAPI staining indicates that exophers do not have nuclear-like DNA content (Fig. 1b), but mitochondrial matrix-localized GFP signals (Fig. 1b) and lysosomal membrane-localized GFP::LMP-1 indicate that mitochondria and lysosomes can be extruded in exophers. EM data also suggest that rough ER material might also constitute cargo of some exophers. It should be noted that multiple entities, i.e., aggregates, mitochondria, and lysosomes, can be included in the same exopher. Thus, if contents are all trash, diverse forms of garbage appear to be collected in the same place before being thrown out together in one large garbage bag, the exopher.

## What are mitochondria doing in exophers?

Functional mitochondria might be needed in exophers to supply energy for remote degradation within the exopher compartment, a possibility raised by the maintenance of networked mitochondria in some exophers. On the other hand, not all exophers include detectable mitochondria (roughly half do), suggesting that mitochondria are not required to power exopher functions after leaving the mother cell.

The obvious question is whether the mitochondria that are extruded might, like aggregates, constitute garbage that could be deleterious to keep around. Indeed, knockdown of transcripts encoding proteins integral to mitochondrial quality control, such as the mitophagy mediator *dct-1*/BNIX3, Parkinson’s disease (PD) genes *pink* and parkin (*pdr-1*), and the mitochondrial unfolded protein response (mUPR) gene *ubl-5*, increase the number of exophers produced by proteo-stressed neurons, suggesting that poor mitochondrial quality is linked to elevated extrusion. Consistent with this idea, redox-sensitive mitochondrial matrix-localized reporter mitoROGFP suggests preferential sorting of mitochondria with elevated matrix oxidation to exophers, while the neurons’ less oxidized counterparts are retained in the soma. Still, the question of mitochondrial functionality, which might itself be impacted under proteo-stress, needs to be addressed in greater depth before a clear understanding of the “rules” for mitochondrial extrusion is accomplished. Overall, though, indications to date suggest that compromised mitochondria might be preferentially extruded from neurons, potentially joining mitophagy as a significant mitochondrial quality control option.

## What are lysosomes doing in exophers?

Lysosomes are degradative organelles critical for autophagy, the degradation of proteins and organelles via acid hydrolases that work in the acidic lysosomal environment. Interestingly, when lysosomes tagged with lysosomal membrane associated protein LMP-1::GFP were tracked, we noted that lysosomes appear included in some, but not all, exophers [1]. Fates of exopher lysosomes seem likely to be different from lysosome exocytosis associated with secretion and membrane repair, in which the lysosome fuses to the plasma membrane to release its contents into the extracellular environment, although more detailed study could change that perception.

What are those lysosomes doing? One possibility is that ejected lysosomes are dedicated to digesting whatever they can within the exopher compartment; another possibility is that defective lysosomes, filled and/or incapacitated by wear and tear, might themselves constitute garbage thrown out for remote degradation. It is clear that a pressing issue is to determine the functional “quality” of lysosomes eliminated in exophers. Genetic elimination of core lysosomal function in the sending neuron will reveal the extent to which sender or receiver (more on that below) contribute to degradation of exopher contents.

## Can neurons throw out debris at any time or is there a scheduled trash day?

A temporal survey of when touch neurons produce exophers during adult life suggests that there may be a trash disposal/pickup schedule—there is a peak of exopher production in early adult life (day 2/3), then exopher levels remain low throughout most of the *C. elegans* reproductive life, but elevate again in late adult life (day 9/10; the reference *C. elegans* wild-type strain N2 lifespan is ~ 21 days). The late-life increase would be consistent with enhanced proteostasis challenges anticipated for aging in the chaperone, proteasome, and autophagy networks. Thus, in older age, throwing out the trash may reflect the last line of defense against accumulating cellular garbage, with a threshold level of material possibly reached.

The “need” for the early-life peak of exopher production during the period of maximum reproduction is more perplexing. Interestingly, though, the early adult extrusion peak coincides with a documented young adult proteostasis reconfiguration from a heavier reliance on the chaperone pathway during development to a heavier reliance on proteasome-mediated degradation in adults [5]. Dye-filled neurons also exhibit the early adult peak of exopher production, consistent with the idea that native stress increases in some neurons during this timeframe. Cells may accumulate trash during development and, as a natural phase in the transition to adult life, dispose of it on a “scheduled” collection day. The importance of the early adult extrusion for reproductive success or longevity awaits experimental testing, as does the distinction between models in which: 1) neurons reach a critical garbage threshold to activate extrusion; or alternatively, 2) there is a temporal “licensing” of the molecular machinery needed for the exopher production event at a particular time of adult development.

## How exactly does an exopher form?

Much remains to be learned about how cellular trash is recognized, localized, sequestered, and ejected, although it is clear that dramatic mobilization of some cell contents must be a part of the story. Accordingly, it is highly likely that specific cytoskeletal proteins and motors will play roles. To the eye, the process of aggregate collection and concentration in a region of the soma is reminiscent of the process of aggresome formation described in mammalian cells, in which aggregates transit down microtubules to a pericentriolar trash collection site prior to attempted degradation [6], a thematic similarity that might suggest candidate required genes that can be tested in genetic screens for impact on exophergenesis.

Further, trash collection appears to occur in one domain of the touch neuron soma and extrusion is often in a particular direction relative to the neuronal process, suggesting some polarity functions might be involved. Indeed, to date, roles for two embryonic polarity genes, *pod-1* (an F-actin binding protein homologous to human coronin 7) and *emb-8* (an NADPH cytochrome 450 reductase involved in lipid production), have been determined to be required for efficient production of exophers in adult neurons under protein aggregation stress. It is absolutely clear that these gene identifications represent only the tip of the iceberg as a large number of genetic activities are certain to be involved in exopher production. Thus, the field can look forward to extensive coupling of gene identification and cell biological studies in the future. When and where modifiers act, and the detailed cellular phenotypes that result when these genes are disrupted, will provide much needed understanding of the operative molecular mechanisms.

## What are those peculiar tubes that appear to connect soma and exopher?

After the exopher buds off the neuronal soma, a thin “string” often appears to connect the sending soma and the nascent exopher (Fig. 1b shows an example). This structure, at least transiently, is an open tube that permits continued accumulation of mCherry in the extruded exopher, as well as transfer of intracellular calcium; mitochondria and lysosomes have also been spotted within these tubes. The tube is ultimately severed, enabling the released exopher to transit through the neighboring hypodermis (see below). The mechanism of breaking, which might involve both mechanical stresses and pinchase machinery provided by the hypodermis, is unknown.

The exopher-associated tubes bear a striking resemblance to tunneling nanotubes (TNTs), which were initially defined as open-ended channels mediating membrane continuity between connected cells and have been observed in bacteria, plant, and animal cells. Tunneling nanotubes contain filamentous actin and, in addition to other roles, have been reported to transport prions, polyQ, alpha synuclein, lysosomes, and mitochondria to neighboring cells [7]. The prominent connecting tubule stage during exopher maturation suggests that a tunneling nanotube might serve as a garbage chute during exophergenesis. Since TNTs have not been widely studied in vivo, the *C. elegans* model now offers the opportunity for extensive genetic dissection of their formation and functions.

## Where does the exopher go?

**Fig. 3.**
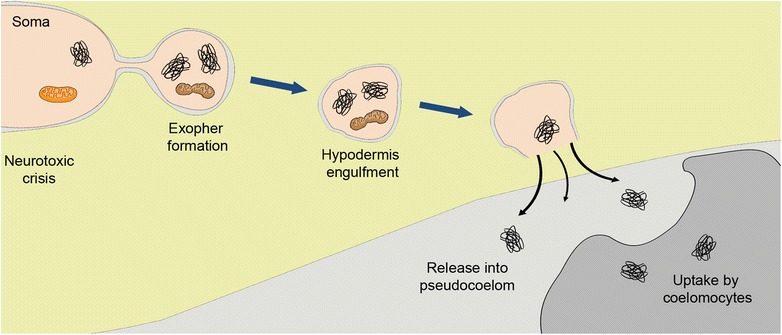
The egress of the exopher. Touch neuron exophers, often including protein aggregates and damaged mitochondria, are extruded into the surrounding hypodermis. In the hypodermis, some exopher contents (possibly mitochondria) can be degraded. However, undegradable contents like mCherry aggregates can be re-extruded from the hypodermis into the body cavity (pseudocoelom). Distant scavenger cells called coelomocytes can take up mCherry from the body cavity, and the mCherry remains within the coelomocytes for the rest of adulthood—possibly analogous to the garbage final resting place—a cellular landfill dump

The egress of the extruded exopher can be followed in vivo as fluorescently tagged aggregates move within the transparent *C. elegans* body (summarized in Fig. 3). *C. elegans* anatomy has been described in exquisite detail such that a nearly complete electron micrographic reconstruction of the young adult body is available (see WormAtlas, http://www.wormatlas.org). The six *C. elegans* touch sensory neurons are located at distinctive positions in the body, lying largely separated from most of the nervous system except when they enter the nerve ring or nerve cord to synapse onto interneurons—their body positions thus enable easy viewing of labeled extrusions around the touch neuron process and soma. With regard to the potential fate of exophers extruded from touch neurons, it is important to consider the fact that the exophers have limited options—the touch neurons are fully embedded in the hypodermis and therefore do not have any direct access to circulating body fluids within the body cavity (pseudocoelom). Thus, exophers can really only exit into the hypodermis.

The exopher enters the hypodermal domain, breaks off the thin connection from the soma, and can appear to “fragment” within the hypodermis. Although relatively few details are known about the exopher’s reception in the hypodermis, cell biological observations and existing EM data suggest a vigorous reaction and indicate that some exopher contents are recognized by hypodermal lysosomes that attempt, and at least partially accomplish, their degradation.

The handling of the invading exopher appears partially dependent upon one specific branch of the conserved *C. elegans* phagocytosis pathway that recognizes apoptotic corpses. This pathway includes the CED-1/DRAPER phagocytosis receptor, CED-6 adaptor, and CED-7/ABC transporter.

This pathway is particularly interesting because in a fly model in which an aggregating polyQ expansion-tagged protein was expressed in specific brain neurons, transfer of large aggregates to neighboring glia occurs via a mechanism that requires the *C. elegans* CED-1 phagocytosis homolog Draper [8], raising the possibility of conservation in the mechanism of aggregate transfer. Further evidence of conservation is suggested by the fact that related human microglial phagocytosis receptor TREM2 is an Alzheimer’s disease risk factor. The process of neighborly removal of cell contents or structures may overlap with the biology of dendritic pruning, given that in *Drosophila* Draper/CED-1 and CED-6 are needed for developmental pruning of outgrowth in mushroom body neurons.

## What happens to the neuronal garbage that is thrown out?

Some mito-GFP tagged structures in exophers appear compacted when they enter the hypodermis, and fluorescent signals dim over time, suggesting the neuronal mitochondria are degraded within the hypodermal compartment. Transcellular degradation would be consistent with *C. elegans* EM data that reveal a vigorous autophagy-like response to the invading exopher. There is precedent for transcellular mitochondrial degradation in mammals; in mouse optic nerve, the retinal ganglion axons shed large exopher-sized vesicles. These vesicles contain acidified mitochondria for internalization and degradation by the lysosomes in neighboring astrocytes. This is described as the primary mode of mitochondrial turnover [9]. A fascinating question, relevant across species, is how the invading vesicle and its contents are recognized for degradation. It might be worth adding that healthy mitochondria have also been shown to be transferred via TNT-like structures into compromised cells to restore their mitochondrial functionality [10]. For better or for worse, mitochondria might be more mobile in biology than currently appreciated.

## What is the fate of the extruded aggregates—are they degraded?

Considerable evidence suggests that some cellular materials, such as large aggregates, cannot be degraded by any cells. Why should the hypodermal lysosomes be any more adept at this degradative task than the neuronal lysosomes? That is to say, an aggregate is expected to be a problem for any cell’s degradative apparatus. So what happens to the mCherry aggregates in the hypodermis? It appears that these aggregates are thrown away again, as mCherry signals disappear from the hypodermis and reappear a few days later in the distant coelomocytes (Fig. 3). The *C. elegans* coelomocytes are large scavenger cells that are generally located in pairs, distributed at three positions in the body. Coelomocytes are known to take up dyes, yolk proteins, and general debris that can be deposited into the pseudocoelom. In animals in which touch neurons express aggregating mCherry and produce exophers at adult day 2/3, red signal is found to concentrate in the distant coelomocytes a few days later. This material must originally come from the touch neuron rather than being inappropriately produced in the coelomocytes because genetic blocking of coelomocyte uptake capacity causes red signal to hang out around the body in a dispersed pattern referred to as “starry night”. Particulate-looking red material is outside the touch neuron, unable to be absorbed and concentrated by coelomocytes. Interestingly, the coelomocyte appears to correspond to the end game—red materials can persist there for the life of the animal. At least for non-digestible mCherry, the coelomocyte is the *C. elegans* trash dump/landfill.

## Exopher production appears to remove so much material from the neuron—this can’t be good for the neuron, can it?

The production of an exopher appears to involve the elimination of a considerable amount of cell content. Still, if only trash is eliminated as data currently suggest, cleaning house is likely a good thing. Consistent with that idea, exopher production from touch neurons does not elicit death of the sending neuron, or dysfunction of the neuronal circuit for touch sensation. In blinded studies with touch neurons sensitized for early onset neuronal dysfunction because of expression of a neurotoxic HuntintinQ128CFP transgene, animals in which an exopher was produced by the stressed neurons retain better touch sensitivity than animals in which the touch neurons did not make them. This finding suggests that producing an exopher is neuroprotective for polyQ-stressed neurons. Still, more targeted analysis of the physiological status of exopher-producing neurons themselves—for example, using cell-specific neuronal activity reporters like calcium sensor GCaMP—will more accurately report on neuronal health post-exopher production. Likewise, whether there are distinctions between young neurons that make exophers and old neurons that make exophers is highly relevant to questions of how exophers might help or hurt aging neurons or aging animals.

## How important is this likely to be?

Given that experimental data generated in diverse species indicate that potentially dangerous aggregates can escape from stressed neurons, it is exciting that *C. elegans* neurons have now been caught in the act of junk selection and extrusion. The exopher discovery in the *C. elegans* model opens up the possibility for rapid genetic and cell biological mechanistic dissection of the associated biology. Understanding of the basic biology of trash expulsion is likely to be important on multiple levels. For one thing, exophergenesis might constitute a previously unappreciated component of cellular quality control, with broad relevance to cellular health: exopher formation might join mitophagy as a complementary strategy for mitochondrial turnover; likewise, the elimination of protein aggregates, rather than sequestration or elimination/reduction via their internal degradation systems, might be a particularly critical facet of proteostasis. Moreover, it is plausible that an exopher-like mechanism underlies the transfer of toxic species in human neurodegenerative disease. Regulated or induced exophergenesis might be developed for novel neuroprotective therapies. Dysfunction of exopher biology might be a critical aspect of disease susceptibility. With these ideas in mind, it will be exciting to evaluate how homologs of human genes implicated in neurodegenerative disease impact *C. elegans* exophergenesis and, conversely, how genes defined as important in *C. elegans* exopher biology impact human neurodegeneration. To address speculation on exopher significance, increased research on cellular garbology (garbology = the study of modern human refuse and trash disposal approaches) will be needed.
